# Long Noncoding RNA TRPM2-AS Promotes the Growth, Migration, and Invasion of Retinoblastoma via miR-497/WEE1 Axis

**DOI:** 10.3389/fphar.2021.592822

**Published:** 2021-04-12

**Authors:** Aipeng Li, Jingpu Yang, Ting Zhang, Lin Li, Miyang Li

**Affiliations:** ^1^ Department of Ophthalmology, The First Hospital of Jilin University, Changchun, China; ^2^ Department of Otolaryngology-Head and Neck Surgery, The Second Hospital of Jilin University, Changchun, China; ^3^ Department of Abdomen Ultrasound, The First Hospital of Jilin University, Changchun, China; ^4^ Department of Ophthalmology, China-Japan Union Hospital of Jilin University, Changchun, China; ^5^ Department of Clinical Laboratory, China-Japan Union Hospital of Jilin University, Changchun, China

**Keywords:** long noncoding RNA, TRPM2-AS, retinoblastoma, miR-497, WEE1

## Abstract

Long noncoding RNAs (lncRNAs) exhibit vital roles in many types of cancer, including retinoblastoma (RB), the most common primary intraocular malignancy tumor of infancy. A novel lncRNA TRPM2-AS has been demonstrated to be related to multiple cancers; however, its role in RB remains unclear. Here, we aimed to investigate the function of TRPM2-AS in RB. In this study, TRPM2-AS expression in 35 human RB tissues and RB cell lines was detected by real-time PCR. And, the relationship between its expression and clinicopathological characteristics of RB patients was analyzed. RB cells’ proliferation, migration, invasion, apoptosis, and cell cycle were explored after silencing TRPM2-AS. The mechanism of TRPM2-AS in RB was focused on miR-497/WEE1 axis. Additionally, the role and mechanism of TRPM2-AS were confirmed in a xenograft mouse model. We found TRPM2-AS expression was enhanced in RB tissues and cells. And, higher TRPM2-AS expression was related to advanced clinical stage and optic nerve invasion in patients. Downregulation of TRPM2-AS significantly inhibited proliferation, migration, and invasion, elevated apoptosis, attenuated G2/M phase arrest in RB cells, and suppressed tumor growth *in vivo*. TRPM2-AS acted as a ceRNA for miR-497 to positively regulate WEE1 expression. miR-497 inhibitor or WEE1 overexpression dramatically reversed the effects of TRPM2-AS downregulating on the malignant phenotypes of RB cells. Therefore, TRPM2-AS is an oncogenic lncRNA in RB, and it functions largely through the miR-497/WEE1 pathway. Despite the limited sample size, this study indicates that TRPM2-AS may be a candidate target in RB therapies.

## Introduction

Retinoblastoma (RB) is the most frequently occurring primary intraocular malignancy tumor of infancy all over the world. It accounts for 6% of cancers in children and with an incidence of one in 15,000 to 20,000 live births worldwide (Dimaras et al., 2015; Fabian et al., 2018). At present, treatment strategies for RB contain surgical enucleation, intravenous chemoreduction and intra-arterial chemotherapy, and external beam radiotherapy (Mendoza and Grossniklaus, 2016). With these treatments, the 5-year survival rate of RB patients has greatly improved. However, the mortality of RB is still as high as 70% in developing countries because of the occurrence of advanced and metastatic tumors, accounting for approximately 9–11% (Balmer et al., 2006; Dimaras et al., 2015; Wang et al., 2017a).

Long noncoding RNAs (lncRNAs) are a kind of noncoding RNAs longer than 200 nucleotides that possess limited protein-encoding ability. Studies have demonstrated that lncRNAs are vital regulators participating in multiple biological and pathological processes, including the formation and progression of cancers (Chen et al., 2020; Huang et al., 2020; Wu and Kuo, 2020). In recent years, accumulating evidence has illustrated that aberrant expression of lncRNAs is associated with the malignant phenotypes and development of RB. For example, the expression levels of small nucleolar RNA host gene 14 (SNHG14) were elevated in RB tissue samples and cell lines. SNHG14 overexpression was involved in the malignant progress in RB patients. And, patients with higher expression of SNHG14 exhibited shorter overall survival than those with lower expression of SNHG14 (Sun et al., 2020). Decreasing SNHG14 inhibited RB cell proliferation, migration, and invasion, promoted apoptosis *in vitro*, and suppressed tumor growth in mouse tumor xenograft models (Sun et al., 2020). The lncRNA TP73-AS1 expression was upregulated in RB tissues and cells and it had oncogenic functions in RB (Wang et al., 2020). Antisense noncoding RNA in the INK4 locus (ANRIL) was also elevated and acted as onco-lncRNA in RB (Wang et al., 2019b; Yu et al., 2019).

TRPM2 antisense RNA (TRPM2-AS) is a novel 875 bp lncRNA located in chr21q22.3 (Pan et al., 2020). It has been demonstrated to be involved in many tumors. TRPM2-AS could promote the progression and radioresistance of gastric cancer (Huang et al., 2019; Xiao et al., 2020). Pan et al. (Pan et al., 2020) found that TRPM2-AS inhibited the proliferation of colorectal cancer. TRPM2-AS was overexpressed in breast cancer cells and downregulation of its expression suppressed the proliferation yet facilitated apoptosis of breast cancer cells (Sun et al., 2019). And, downregulation of TRPM2-AS inhibited the resistance of non-small-cell lung cancer cells to cisplatin (Ma et al., 2017). However, the role and underlying mechanism of TRPM2-AS in RB remain unclear. In this study, we explored the expression level of TRPM2-AS in human RB tissues and cells. The function roles and possible mechanism of TRPM2-AS were investigated through downregulation of its expression levels in RB cells.

## Materials and Methods

### Patients

A total of 35 RB specimens from patients who underwent an ophthalmectomy at the First Hospital of Jilin University, the Second Hospital of Jilin University, and the China-Japan Union Hospital of Jilin University were included in this study. These enrolled patients were 19 males and 16 females, aged between 5 months and 8 years old, and they did not receive any clinical treatment before admission. Nine normal retina tissues collected from patients with ophthalmorrhexis who underwent enucleation were recruited as the control. Their ages were between 3 months and 9 years. Each sample was verified by pathological diagnosis in a blind-manner. Following surgery, fresh tissues were immediately frozen in liquid nitrogen and stored at −80°C. Written informed consent was obtained from patients and guardians prior to the study. The present study was approved by the Ethics Committee of The First Hospital of Jilin University (No. 2018-067).

### Cell Culture

The retinoblastoma cell lines Y79 and Weri-Rb1 purchased from ATCC (Rockville, MD, United States) were used, and ARPE-19 human retinal epithelial cells were used as the control. Y79 and Weri-Rb1 cells were cultured in RPMI 1640 medium (Hyclone, Logan, Utah, United States), including 10% heat-inactivated fetal bovine serum (FBS; Gibco, Grand Island, NY, United States), 100 U/ml penicillin, and 100 μg/ml streptomycin. ARPE-19 cells were maintained in DMEM medium (Hyclone). These cells were all maintained in an incubator set at 37°C with 5% CO_2_.

### Cell Transfection

Three short-hairpin RNAs (shRNAs) targeting TRPM2-AS and the corresponding nontargeting sequences were synthesized by GenePharma (Shanghai, China) and then inserted into a pRNAT-U6.1/Neo plasmid. miR-497 mimic and inhibitor and their corresponding controls were designed and synthesized by GenePharma Co., Ltd. WEE1 cDNA was amplified from human RB tissues and then subcloned into the pcDNA3.1 plasmid. Y79 and Weri-Rb1 cells were transfected with the above vectors using Lipofectamine 2000 (Invitrogen, Carlsbad, CA, United States) according to the manufacturer’s protocol. TRPM2-AS stable knockdown cells were established by G418 selection (400 μg/ml, Sigma-Aldrich, St Louis, MO, United States).

### Cell Proliferation Capacity Analysis

Cell Counting Kit-8 (CCK8; Dojidon, Kumamoto, Japan) assay was used to detect cell proliferation. After transfection for 24 h, 2,000 cells/well were seeded into 96-well plates and cultured at 37 °C with 5% CO_2_. After 24, 48, and 72 h of incubation, 10 μl CCK8 solution (5 mg/ml) was added to the cultured cells. After being cultured at 37°C for another 2 h, a microplate reader was used to measure the optical density (OD) of each well at a wavelength of 450 nm.

### Transwell Assay for Cell Migration and Invasion

We used 24-well transwell chambers to investigate the migration and invasion of RB cells. For invasion assays, transwell chambers were precoated with Matrigel. For each assay, cells (5 × 10^4^) resuspended into a serum-free medium (200 μl) were added to the upper chamber; simultaneously, 600 μl of serum medium was added to the lower compartment. After a 24 h incubation, cells on the upper chamber membrane were wiped off, and the migrated or invaded cells on the bottom sides were fixed with 4% paraformaldehyde and stained with 0.1% crystal violet. An inverted microscope was used to observe stained cells and cell numbers were counted. Six randomized fields were picked.

### Measurement of Cell Cycle and Apoptosis

Flow cytometry was used to detect cell cycle distribution and apoptosis. For cell cycle analysis, cells were harvested, washed with PBS, and fixed in 70% ethanol at 4°C overnight. Then, samples were stained with propidium iodide (PI; Abcam) according to the manufacturer’s protocol. For apoptosis assay, cells were incubated with FITC-conjugated Annexin V and PI for 15 min in the dark at room temperature. Cells were analyzed using the BD FACSCalibur (Becton Dickinson, Mountain View, CA, United States).

### Bioinformatic Predictions and Luciferase Report Assay

Starbase (http://starbase.sysu.edu.cn/) online software was used to predict miRNAs binding to TRPM2-AS and the potential target genes of miR-497. The DNA regions coding wild-type and mutated sequence of TRPM2-AS-containing miR-497 binding sequence were constructed and cloned into the pGL3 luciferase reporter vector. Luciferase reporter plasmids were cotransfected with miR-497/control mimic into Y79 cells using Lipofectamine 2000. Luciferase activities of cells under different treatments were measured using a Dual-Luciferase assay kit (Promega, Madison, WI, United States) at 48 h after transfection. Relative luciferase activity was defined as the ratio of firefly luciferase activity to Renilla luciferase activity.

### Biotinylated miRNA Pull-Down Assay

The biotinylated miR-497 mimic or control mimic (100 nM) synthesized by GenePharma was transfected into Weri-Rb1 cells. Forty-eight hours after transfection, whole cell lysates were harvested by lysing with lysis buffer containing protease inhibitor cocktail and RNase inhibitor. The biotinylated RNA complex was pulled down after incubating the cell lysates with M-280 streptavidin magnetic beads (Invitrogen, Carlsbad, CA, United States) at 4°C overnight. Total RNA was extracted with Trizol reagent (Invitrogen). The abundance of TRPM2-AS and WEE1 in the combined fraction was quantified by real-time PCR.

### Xenograft Mouse Model

Five-week-old female BALB/C athymic nude mice were obtained from the National Laboratory Animal Center (Beijing, China). All mice were grown in specific pathogen-free conditions and allowed free access to food and water. After 1 week of accommodation, the animals were randomly divided into TRPM2-AS shRNA group and control shRNA group (*n* = 5 per group), in which each mouse was injected subcutaneously in the right flank with 1 × 10^7^ Weri-Rb1 cells stably knockdown of TRPM2-AS or its control. Tumor size was monitored using calipers every 4 days and tumor volume was measured with the following formula: V = 1/2 × (longest diameter) × (shortest diameter)^2^. Mice were euthanized by cervical dislocation at 28 days after implantation. Tumor tissues were excised for further study. All animal experiments were performed in accordance with the Animal Ethics Committee of the First Hospital of Jilin University (No. 2018-067).

### Real-Time PCR

Total RNA was extracted from tissues and RB cells using the Trizol reagent (Invitrogen). RNA concentration was detected at 260 nm using a spectrophotometer, with an OD 260/280 ratio of 1.8-2.0 used only. For TRPM2-AS and WEE1 assay, isolated RNA was reversely transcribed into cDNAs with PrimeScriptTM RT-PCR Kit (TaKaRa, Dalian, China). Real-time PCR was carried out using SYBR® Premix Ex Taq (Takara). For miR-497 analysis, reverse transcription was conducted using PrimeScriptTM miRNA RT-PCR Kit (TaKaRa). The relative expression of the gene was calculated based on the 2^−ΔΔCt^ method, with GAPDH or U6 as the internal references. The primer sequences were shown as follows: TRPM2-AS forward 5′- TGC​GGT​TAC​GAG​GGC​AAA​TA-3′, reverse 5′-CAC​GTA​GGC​TGA​GTG​ACG​AG-3’; WEE1 forward 5′-TGC​ACT​TGT​CTT​TGA​CTT​GTG​TTT-3′, reverse 5′-ATC​AAA​ATG​TGC​CCT​CTG​CTT​T-3′; GAPDH forward 5′- CGG​ATT​TGG​TCG​TAT​TGG​GC-3′, reverse 5′-CCC​GTT​CTC​AGC​CAT​GTA​GTT-3′; miR-497 forward 5′-ACA​CTC​CAG​CTG​GGC​AGC​AGC​ACA​CTG​TG-3′, reverse 5′-TGG​TGT​CGT​GGA​GTC​G-3′; U6 forward 5′-CTC​GCT​TCG​GCA​GCA​CA-3′, reverse 5′-AAC​GCT​TCA​CGA​ATT​TGC​GT-3′.

### Western Blot

Total proteins from RB cells and tissues were isolated by RIPA lysis buffer and the concentrations of proteins were quantified using a BCA kit (Thermo, Carlsbad, CA, United States). Equal amounts of protein extractions were separated by SDS-PAGE and transferred onto PVDF membranes. After washing with TBST, the membranes were incubated with diluted primary antibodies (all were from Cell Signaling Technology, Danvers, MA, United States), including anti-Bcl-2, anti-Bax, anti-cleaved caspase 3, anti-WEE1, anti-cyclin B1, anti-cyclin-dependent kinase 1 (CDK1), and anti-p-CDK1 (Tyr 15) overnight at 4°C, followed by incubation with an HRP-labeled secondary antibody for 1 h at room temperature. Subsequently, the membranes were washed with TBST and enhanced chemiluminescence-Plus reagent (GE Healthcare, Little Chalfont, United Kingdom) was added to visualize signals. GAPDH was used as a loading control.

### Statistical Analysis

Experimental data are expressed as the means ± standard deviation (SD), and all experiments were performed in triplicate. All analyses were carried out using GraphPad Prism 7.0 software (GraphPad, Inc.). The one-way ANOVA test or Student’s *t*-test was used to compare differences between groups. *p* < 0.05 was considered statistically significant.

## Results

### TRPM2-AS Is Increased in Human RB Tissues and Cells

**FIGURE 1:**
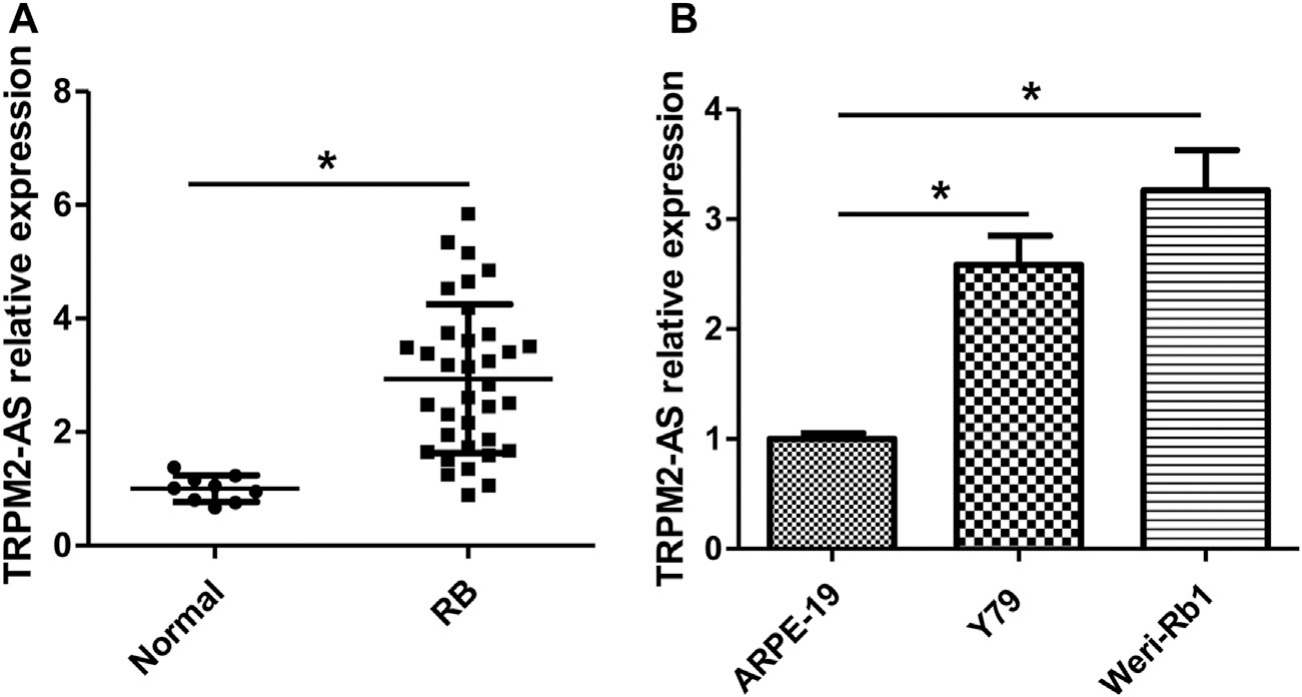
TRPM2-AS expression is increased in human RB tissues and cells. **(A)** Real-time PCR was used to measure the relative expression of TRPM2-AS in 35 RB and nine normal tissue samples. **(B)** The relative expression of TRPM2-AS was higher in Y79 and Weri-Rb1 cells than ARPE-19 cells. *p < 0.05.

Levels of TRPM2-AS expression in human RB tissues were analyzed by real-time PCR, and the results showed that TRPM2-AS expression was higher in RB tissues than normal retinal tissues (Figure 1A). According to the median value of TRPM2-AS, RB samples were assigned into TRPM2-AS low- or high-level group. High expression of TRPM2-AS was associated with advanced clinical stage and optic nerve invasion. On the other hand, TRPM2-AS expression was not notably related to age, gender, laterality, differentiation, and family history of patients (Table 1). The expression of TRPM2-AS was also detected in RB cells. We found that compared with nontumor cell line ARPE-19, TRPM2-AS expression levels were upregulated in RB cell lines, including Y79 and Weri-Rb1 (Figure 1B).

**TABLE 1:** Correlation between expression of TRPM2-AS/WEE1 and clinicopathological factor in RB patients.

Clinicopathological features	Number	TRPM2-AS			WEE1		
		Low (18)	High (17)	*p* value	Low (17)	High (18)	*p* value
Age				0.146			0.471
<3	25	15	10		11	14	
≥3	10	3	7		6	4	
Gender				0.505			0.738
Male	19	11	8		10	9	
Female	16	7	9		7	9	
Laterality				0.402			0.691
Unilateral	28	13	15		13	15	
Bilateral	7	5	2		4	3	
Clinical stage				0.018^*^			0.007^*^
Early stages (A, B)	9	8	1		8	1	
Advanced stages (C, D, E)	26	10	16		9	17	
Differentiation				0.50			0.092
Well/moderately	15	9	6		10	5	
Poorly/undifferentiated	20	9	11		7	13	
Optic nerve invasion				0.002^*^			0.035^*^
Negative	22	16	6		14	8	
Positive	13	2	11		3	10	
Family history				0.603			0.229
Negative	32	17	15		17	15	
Positive	3	1	2		0	3	

*p <0.05.

### Downregulation of TRPM2-AS Decreases RB Cell Proliferation, Migration, and Invasion, Promotes Cell Apoptosis, and Attenuates G2/M Phase Arrest

**FIGURE 2:**
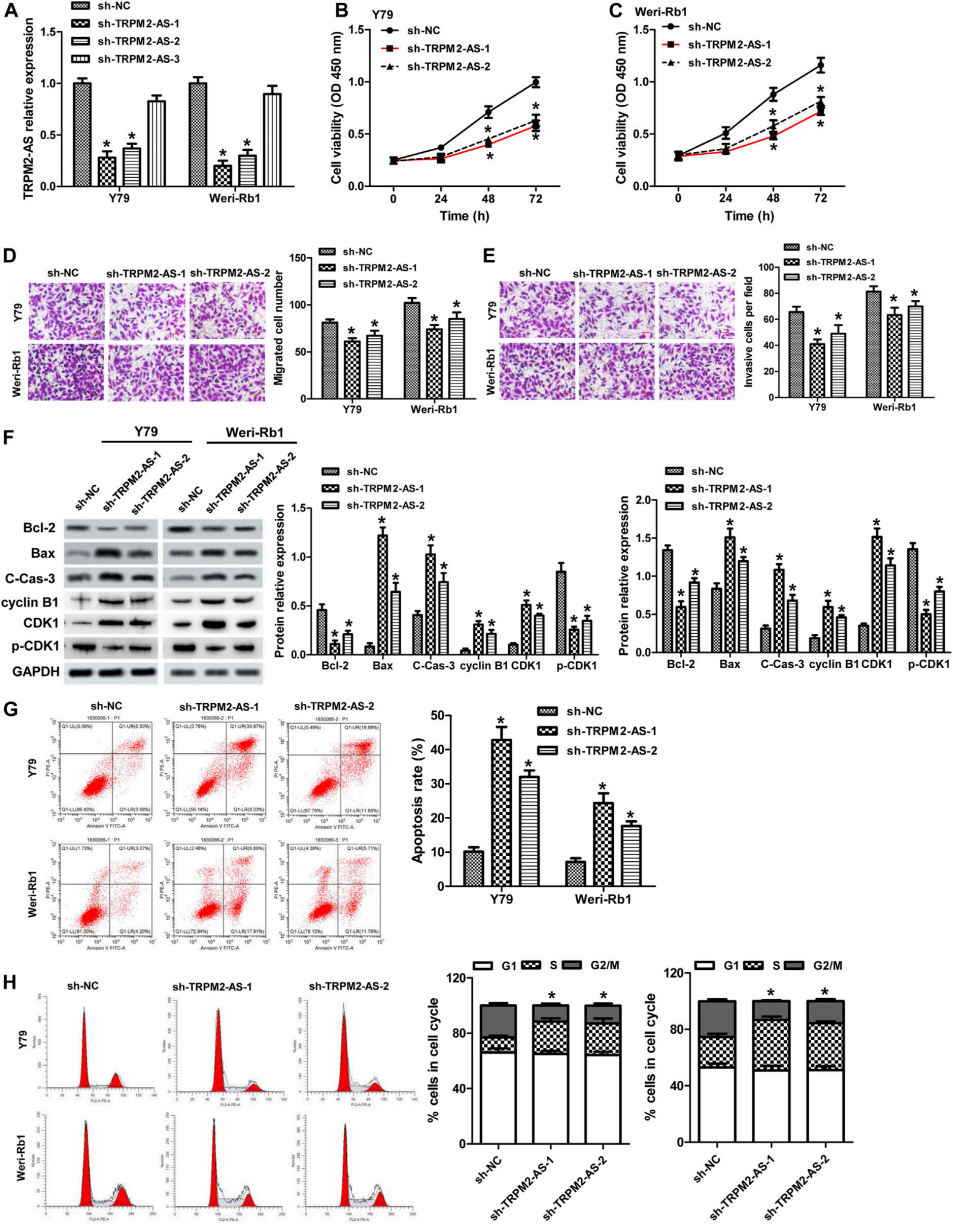
Downregulation of TRPM2-AS decreases RB cell proliferation, migration, and invasion, promotes cell apoptosis, and attenuates G2/M phase arrest. **(A)** Y79 and Weri-Rb1 cells were transfected with three shRNAs targeting TRPM-AS-1, and the knockdown efficiency was examined by real-time PCR. CCK-8 assay for the proliferation of Y79 **(B)** and Weri-Rb1 **(C)** cells. **(D)** Migration of both RB cells was inhibited by TRPM-AS-1 shRNA. **(E)** The invasion of RB cells was determined by transwell. **(F)** Protein levels of Bcl-2, Bax, cleaved caspase-3 (C-Cas-3), cyclin B1, CDK1, and p-CDK1 in Y79 and Weri-Rb1 cells were analyzed by western blot. **(G)** Flow cytometry assay for the apoptosis of cells. **(H)** Cell cycle distribution of Y79 and Weri-Rb1 cells was examined by flow cytometry. *p < 0.05 vs. the sh-NC group.

The function of TRPM2-AS in RB was evaluated by knockdown of TRPM2-AS in Y79 and Weri-Rb1 cells. Three shRNAs targeting TRPM2-AS were introduced into these cells. The knockdown efficiency was examined by real-time PCR, and the results revealed that sh-TRPM2-AS-1 and sh-TRPM2-AS-2 were more efficient in reducing TRPM2-AS expression (Figure 2A). These two shRNAs were used in subsequent loss-of-function assays. The proliferation of Y79 and Weri-Rb1 cells was significantly decreased after TRPM2-AS silencing in Y79 and Weri-Rb1 cells (Figures 2B,C). Transwell assay was carried out to detect cell migration and invasion and it was indicated that the migrative and invasive capacities of Y79 and Weri-Rb1 cells were both suppressed after knockdown of TRPM2-AS (Figures 2D,E). To confirm whether the proliferation of Y79 and Weri-Rb1 cells could affect cells’ migrative and invasive capacities, CCK-8 was used to analyze the viabilities of Y79 and Weri-Rb1 cells in the serum-free medium. Through the growth curve of RB cells, we found that the viabilities of Y79 and Weri-Rb1 cells were not affected by sh-TRPM2-AS transfection in the serum-free medium (Supplementary Figure S1). Cell apoptosis was measured by proteins related to apoptosis and confirmed by flow cytometry. The results showed that Bcl-2 expression was reduced, levels of Bax and cleavage of caspase-3 were enhanced (Figure 2F), and the number of apoptotic cells was increased after TRPM2-AS downregulation (Figure 2G). Additionally, the effect of TRPM2-AS silencing on the cell cycle was determined and the results revealed that knockdown of TRPM2-AS significantly attenuated G2/M phase arrest (Figure 2H). And, the levels of proteins related to G2/M phase were analyzed and the results showed that cyclin B1 and CDK1 were elevated and p-CDK1 was decreased by sh-TRPM2-AS (Figure 2F). Thus, these data indicated that TRPM2-AS silencing decreased the proliferation, migration, and invasion, promoted apoptosis, and attenuated G2/M phase arrest in RB cells.

### TRPM2-AS Directly Binds to miR-497

**FIGURE 3:**
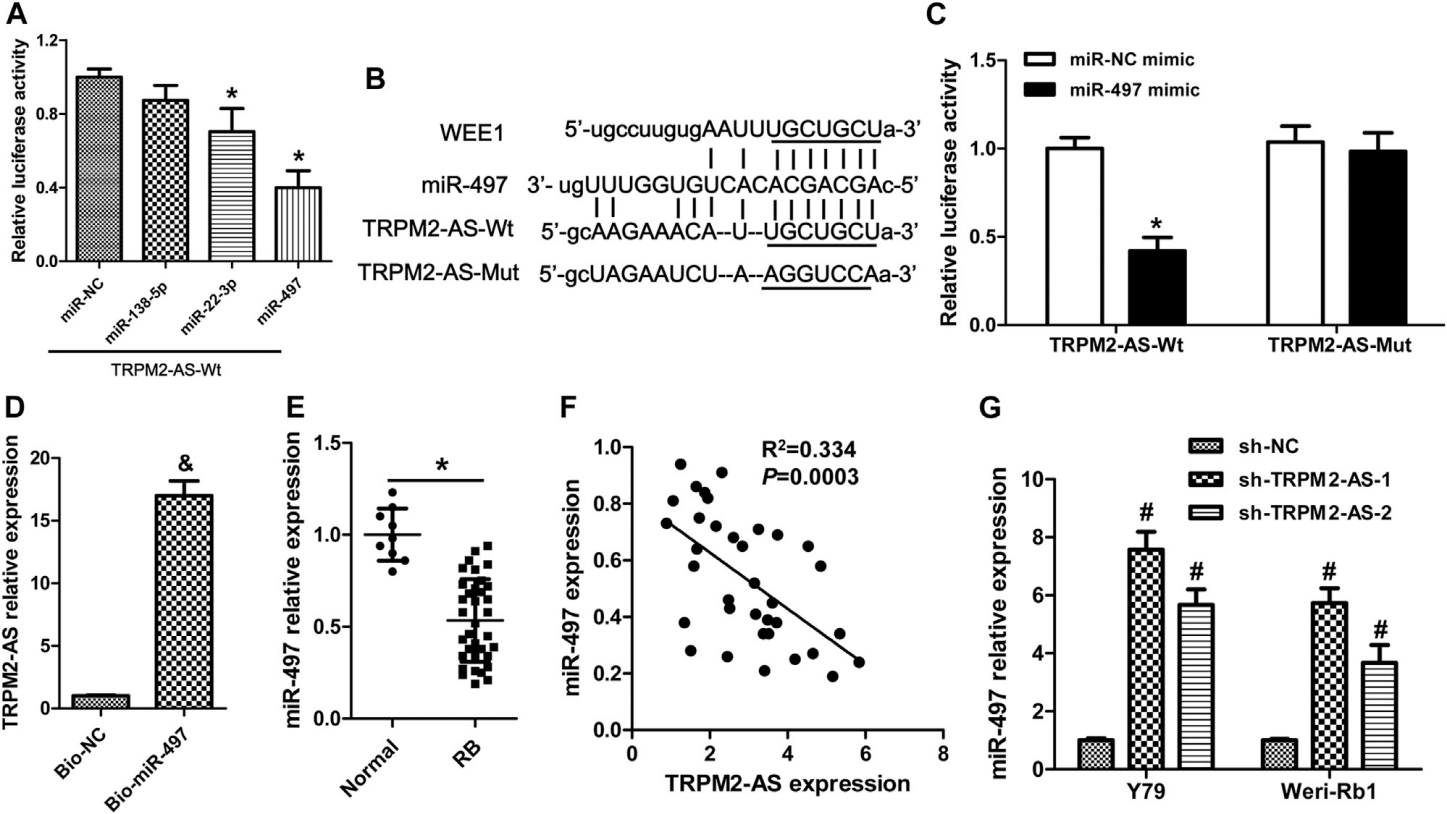
TRPM2-AS directly binds to miR-497. **(A)** Relative luciferase activity from Y79 cells cotransfected with the TRPM2-AS reporter plasmid and three candidate miRNAs. **(B)** The binding sequences between TRPM2-AS/WEE1 and miR-497. **(C)** The relative luciferase activity of Y79 cells cotransfected with pGL3-TRPM2-AS wild/mutant type and miR-497/miR-NC mimic. **(D)** Weri-Rb1 cells were transfected with biotin-labeled (Bio)-miR-497 or its control (Bio-NC); the enrichment of TRPM2-AS in the biotinylated RNA complex was analyzed. **(E)** miR-497 expression in human tissues was detected by real-time PCR. **(F)** Correlation between TRPM2-AS and miR-497 in human RB tissues. (**G)** miR-497 expression was increased in RB cells after downregulation of TRPM2-AS. *p < 0.05 vs. the miR-NC mimic or normal group; ^&^ *p* < 0.05 vs. the Bio-NC group; ^#^ *p* < 0.05 vs. the sh-NC group.

Starbase bioinformatics online software, which was used to predict miRNAs binding to TRPM2-AS, revealed that 13 miRNAs (miR-138-5p, miR-22-3p, miR-497, miR-132-5p, miR-424-5p, miR-6838-5p, miR-16-5p, miR-15b-5p, miR-195-5p, miR-15a-5p, miR-103a-3p, miR-107, and miR-942-5p) may act as biological targets of lncRNA TRPM2-AS. Then, by searching published articles to determine whether these miRNAs have been demonstrated to be related to RB, we found that only miR-138-5p (Wang et al., 2017b), miR-22-3p (Liu et al., 2018), and miR-497 (Li et al., 2017a) matched the criterion. Lastly, we performed a luciferase reporter assay to validate the binding of three candidate miRNAs with TRPM2-AS, and the results showed that miR-497 was the most effective miRNA that reduced luciferase activity of pGL3-TRPM2-AS (Figure 3A). Hence, miR-497 was selected in the following study. The predicted binding sites between miR-497 and TRPM2-AS were shown in Figure 3B. Our results further revealed that miR-497 mimic could not affect the luciferase activity of the mutant plasmid of TRPM2-AS in Y79 cells (Figure 3C). These data indicated that miR-497 could downregulate TRPM2-AS expression. RNA pull-down assay was also used to confirm the direct binding between TRPM2-AS and miR-497. The results indicated that the enrichment of TRPM2-AS was higher in Weri-Rb1 cells transfected with biotin-labeled (Bio)-miR-497 than cells transfected with Bio-NC (Figure 3D). miR-497 expression assay in human tissues revealed that the relative expression of miR-497 was lower in human RB tissues than that in normal retinal tissues (Figure 3E). And, there was a negative correlation between miR-497 and TRPM2-AS in these RB samples (Figure 3F). Additionally, we found that TRPM2-AS knockdown elevated miR-497 expression in Y79 and Weri-Rb1 cells (Figure 3G). Taken together, these results suggested that there was a negative feedback regulation between TRPM2-AS and miR-497.

### miR-497 Inhibitor Attenuates TRPM2-AS Knockdown on RB Malignant Phenotypes

**FIGURE 4:**
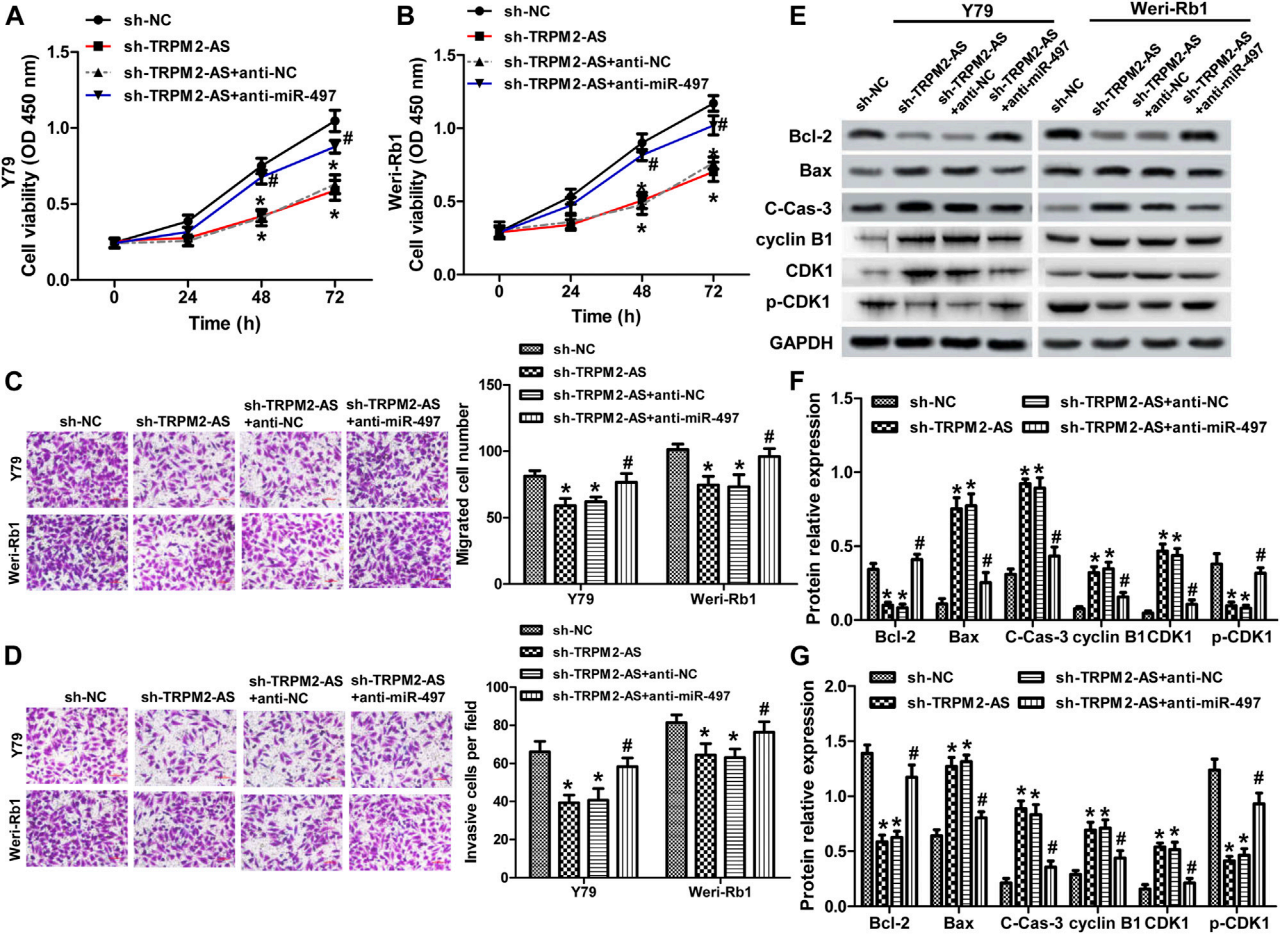
miR-497 inhibitor attenuates TRPM2-AS knockdown on RB malignant phenotypes. Y79 **(A)** and Weri-Rb1 **(B)** cells were transfected with TRPM2-AS/control shRNA with or without miR-497/control inhibitor. And, cell proliferation was determined by CCK-8 assay. The migration **(C)** and invasion **(D)** of both RB cells under different treatments were assayed. **(E–G)** The protein levels of Bcl-2, Bax, cleave-caspase-3, cyclin B1, CDK1, and p-CDK1 were investigated by western blot. *p < 0.05 vs. the sh-NC group; ^#^ *p* < 0.05 vs. the sh-TRPM2-AS group.

Next, we analyzed whether TRPM2-AS functioned in RB via modulating miR-497 expression. A rescue experiment was carried out using a miR-497 inhibitor. The results showed that miR-497 inhibitor abolished the suppressed effects of TRPM2-AS silencing on the proliferation, migration, and invasion of RB cells (Figures 4A–D). And, the protein levels of Bcl-2, Bax, and cleave-caspase-3 altered by TRPM2-AS knockdown were dramatically reversed by miR-497 inhibitor in both RB cells (Figures 4E–G). In addition, the influences of TRPM2-AS downregulation on the protein levels of cyclin B1, CDK1, and p-CDK1 were abrogated by a miR-497 inhibitor (Figures 4E–G). These results indicated that TRPM2-AS functioned via negatively regulating miR-497 expression in RB.

### TRPM2-AS Mediates WEE1 Expression via Regulating miR-497 in RB Cell Lines

**FIGURE 5:**
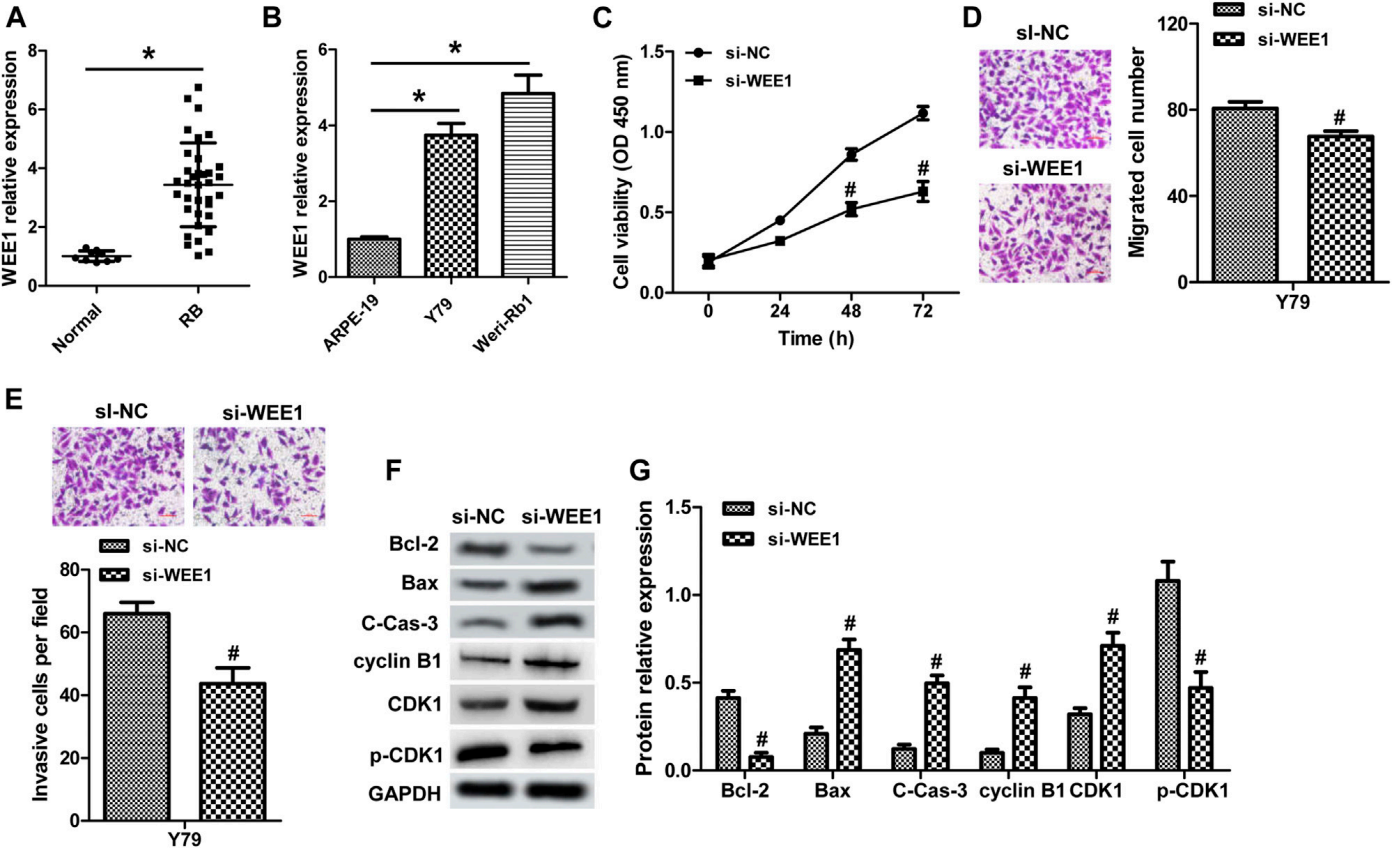
WEE1 promotes the malignant phenotypes of RB. **(A)** WEE1 expression in 35 RB and nine normal tissue samples was detected by real-time PCR. **(B)** The expression of WEE1 in RB cell lines and ARPE-19 cells. **(C)** Y79 cells transfected with WEE1 siRNA showed inhibited cell proliferation compared with the control siRNA group. The migration **(D)** and invasion **(E)** of Y79 cells transfected with WEE1 siRNA or control siRNA were analyzed by transwell. **(F,G)** Western blot assay for apoptosis- and cell cycle–related proteins in Y79 cells. *p < 0.05 vs. the normal group or ARPE-19 cells; ^#^ *p* < 0.05 vs. the si-NC group.

**FIGURE 6:**
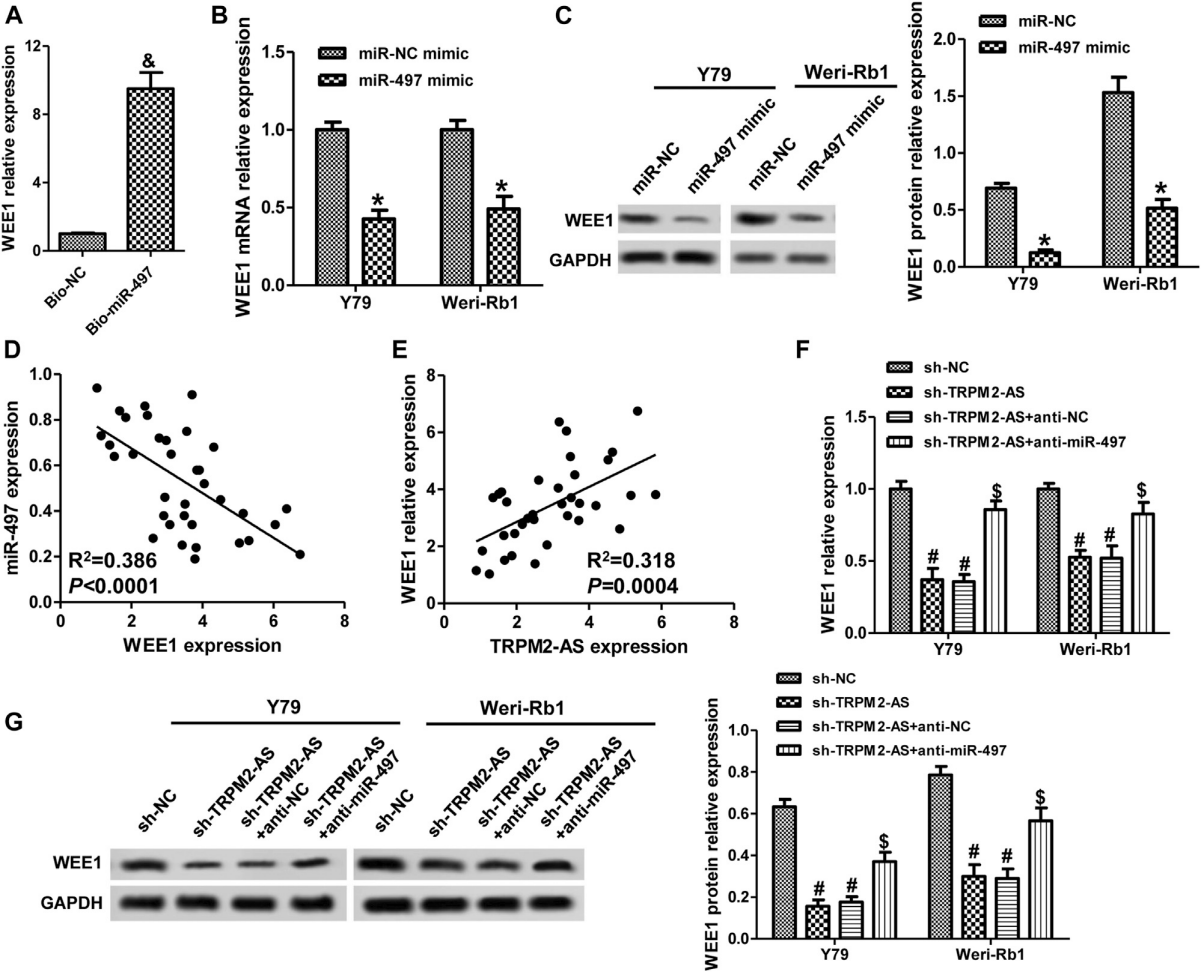
TRPM2-AS mediates WEE1 expression via regulating miR-497 in RB cell lines. **(A)** The abundance of WEE1 in Weri-Rb1 cells transfected with Bio-miR-497 or Bio-NC. Y79 and Weri-Rb1 cells were transfected with miR-497 or control mimic, and the mRNA **(B)** and protein **(C)** levels of WEE1 were measured by real-time PCR and western blot, respectively. **(D)** The connection between WEE1 and miR-497 expression in 35 RB tissues was analyzed by Pearson correlation analysis. **(E)** The correlation between WEE1 and TRPM2-AS in 35 RB samples. **(F)** Y79 and Weri-Rb1 cells were transfected TRPM2-AS shRNA with or without miR-497 inhibitor, and WEE1 mRNA expression was detected by real-time PCR. **(G)** The protein levels of WEE1 were assayed by western blot. ^&^ *p* < 0.05 vs. the Bio-NC group; *p < 0.05 vs. the miR-NC mimic group; ^#^ *p* < 0.05 vs. the sh-NC group; ^$^ *p* < 0.05 vs. the sh-TRPM2-AS group.

WEE1, a member of the Serine/Threonine protein kinase family, is upregulated and possesses oncogenic functions in several cancer types (Li et al., 2017b; Wang et al., 2019a). It has been found to be a target gene of miR-497 in neuroblastoma (Creevey et al., 2013). Currently, the role of WEE1 in RB remains unclear and the association between WEE1 and miR-497 in RB. WEE1 expression was upregulated in human RB tissues and cells (Figures 5A,B). And, elevated WEE1 expression was related to advanced clinical stage and optic nerve invasion in RB patients (Table 1). Decreasing of WEE1 in Y79 cells significantly suppressed cell proliferation, migration, and invasion, promoted the protein levels of Bax, cleaved caspase-3, cyclin B1, and CDK1, and decreased the expression of Bcl-2 and p-CDK1 compared with the control (Figures 5C–G). These data indicated that WEE1 contributed to the development of RB. The RNA pull-down results showed that the enrichment of WEE1 was enhanced in Weri-Rb1 cells transfected with Bio-miR-497 than cells transfected with Bio-NC (Figure 6A). More importantly, miR-497 mimic significantly decreased WEE1 mRNA and protein levels in RB cells (Figures 6B,C), and its level in RB tissues was negatively associated with miR-497 (Figure 6D). Taken together, these results indicated that WEE1 was a target of miR-497 in RB.

Subsequently, we investigated the relationships among TRPM2-AS, miR-497, and WEE1 in RB. In human RB tissues, there was a positive correlation between WEE1 and TRPM2-AS (Figure 6E). Knockdown of TRPM2-AS markedly decreased WEE1 expression, which was abrogated by a miR-497 inhibitor (Figures 6F,G). By comparing the binding sites between TRPM2-AS/WEE1 and miR-497, we found that TRPM2-AS and WEE1 had the same binding sequences on miR-497 (Figure 3B). These results confirmed that TRPM2-AS was a competing endogenous RNA (ceRNA) for miR-497 to regulate WEE1.

### Upregulation of WEE1 Abrogated the Influence of TRPM2-AS Knockdown on RB Cells

**FIGURE 7:**
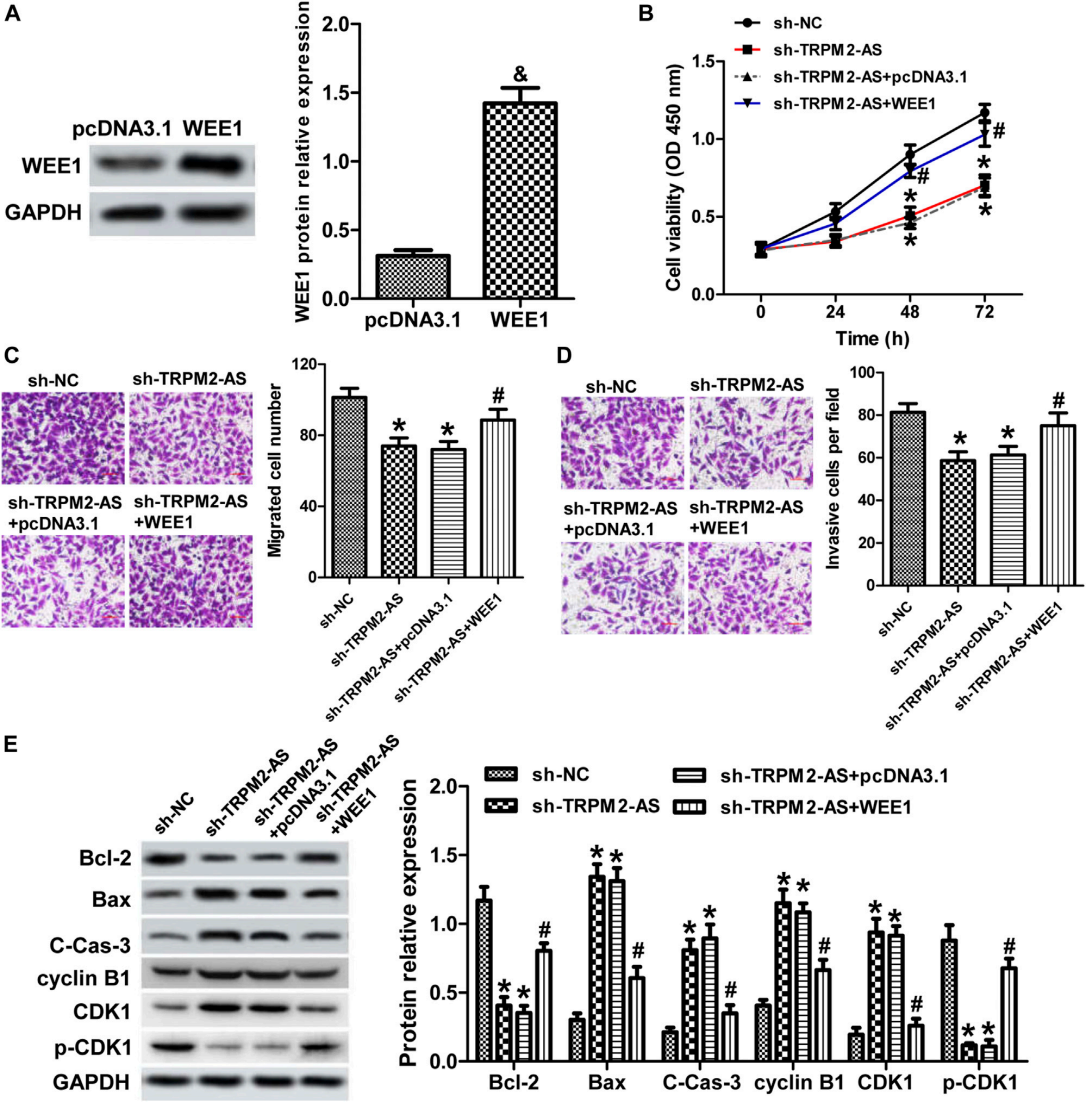
Upregulation of WEE1 abrogated the influence of TRPM2-AS knockdown on RB cells. **(A)** WEE1 protein levels were elevated in Weri-Rb1 cells were transfected with pcDNA3.1-WEE1 plasmid compared with its control plasmid. **(B)** Weri-Rb1 cells were transfected with TRPM2-AS shRNA and pcDNA3.1-WEE1 plasmid. Cell proliferation was analyzed by CCK-8 assay. The migrated **(C)** and invasive **(D)** cell numbers suppressed by TRPM2-AS shRNA were reversed by WEE1 overexpressing. (**E)** Protein levels of Bcl-2, Bax, cleave-caspase-3, cyclin B1, CDK1, and p-CDK1 in Weri-Rb1 cells under different treatments. ^&^ *p* < 0.05 vs. the pcDNA3.1 group; *p < 0.05 vs. the sh-NC group; ^#^ *p* < 0.05 vs. the sh-TRPM2-AS group.

We further verified whether TRPM2-AS functions via mediating WEE1 expression by reintroduction of WEE1 in RB cells with TRPM2-AS knockdown. The protein levels of WEE1 were upregulated in cells transfection with pcDNA3.1-WEE1 plasmid, compared with the control plasmid (Figure 7A). The proliferation, migration, and invasion of Weri-Rb1 cells weakened by TRPM2-AS depletion were strengthened in response to WEE1 upregulation (Figures 7B–D). Overexpression of WEE1 abolished the impacts of TRPM2-AS knockdown on the levels of Bcl-2, Bax, cleaved caspase-3, cyclin B1, CDK1, and p-CDK1 (Figure 7E). Therefore, these findings demonstrated that TRPM2-AS facilitated the malignant characteristics of RB via promoting WEE1.

### TRPM2-AS Knockdown Suppressed Tumor Growth *In Vivo*

**FIGURE 8:**
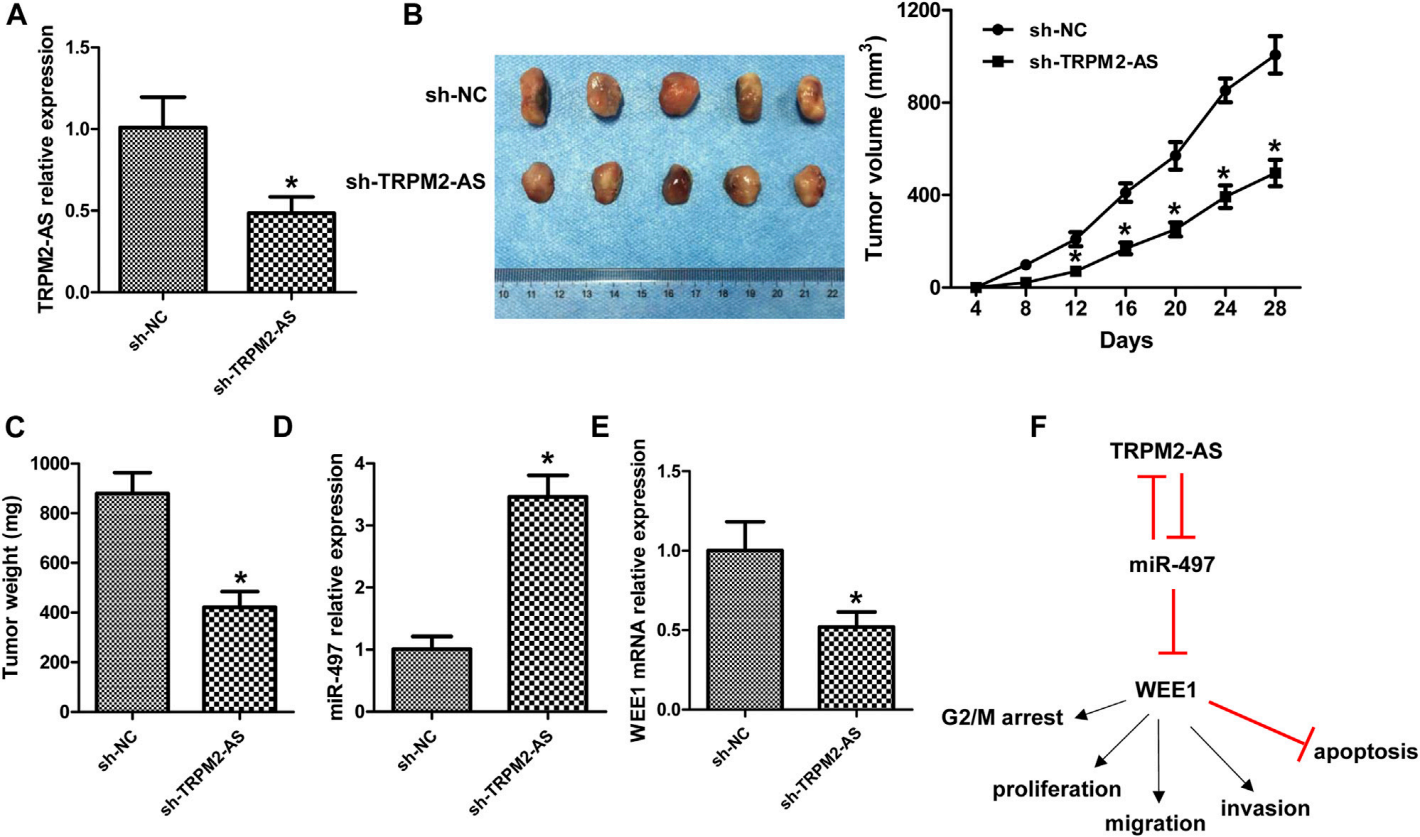
Knockdown of TRPM2-AS suppressed tumor growth *in vivo*. **(A)** TRPM2-AS expression in mouse tumor tissues. **(B)** Representative tumor images and the measurement of tumor volume in mice. **(C)** Tumor weight. The expression of miR-497 **(D)** and WEE1 **(E)** was analyzed in mouse tumor tissues. **(F)** Schematic diagram of TRPM2-AS in the development of RB. N = 5, *p < 0.05 vs. the sh-NC group.

We also evaluated the role of TRPM2-AS in RB in a xenograft mouse model. TRPM2-AS shRNA notably downregulated TRPM2-AS expression in tumor tissues (Figure 8A). Downregulation of TRPM2-AS significantly inhibited tumor growth, as revealed by the suppressed tumor volume and tumor weight (Figures 8B,C). And, the silencing of TRPM2-AS dramatically increased miR-497 expression and decreased WEE1 expression (Figures 8D,E).

## Discussion

Various studies have clarified that gene mutation and changes of gene expression features were closely involved in the initiation and progression of RB (Dimaras et al., 2015; Winter et al., 2020; Zocchi et al., 2020). With the development of molecular techniques, several RB-associated lncRNAs have been reported (Yang and Wei, 2019). In the present study, we demonstrated that the increase of TRPM2-AS in RB could promote cell’s proliferative, migrative, and invasive abilities and elevate G2/M cell cycle arrest, yet suppress cell apoptosis. Molecular mechanism investigation revealed that TRPM2-AS functioned via increasing WEE1 expression through negatively modulating miR-497 expression, and miR-497 could also negatively regulate TRPM2-AS expression (Figure 8F).

In the last several years, the function roles of TRPM2-AS in many cancers have been identified, including gastric cancer, colorectal cancer, breast cancer, and non-small-cell lung cancer (Ma et al., 2017; Huang et al., 2019; Sun et al., 2019; Pan et al., 2020; Xiao et al., 2020). In this study, we found TRPM2-AS expression was higher in human RB tissues than normal retinal tissues. And, higher expression of TRPM2-AS in RB tissues was involved in the advanced clinical stage and optic nerve invasion in patients. Elevated TRPM2-AS expression was also observed in RB cell lines in comparison with nontumor cell lines. These results indicated that TRPM2-AS might be an oncogene in the development of RB. To verify the role of TRPM2-AS in RB, we downregulated TRPM2-AS expression using shRNA technology. Downregulation of TRPM2-AS significantly decreased the proliferation, migration, and invasion of RB cells and tumor growth *in vivo*. Apoptosis, a very tightly programmed cell death, plays a vital role in the development and homeostasis in normal tissues, whereas tumor cells can evade apoptosis, which promotes tumor development and metastasis (Hassan et al., 2014). Tumor cells become apoptosis defects via upregulating expression of antiapoptotic proteins like Bcl-2 or via downregulating levels of proapoptotic proteins such as Bax (Hassan et al., 2014). Apoptosis is a process dependent on caspases, and caspase-3 plays essential roles in the cleavage of the majority of apoptosis-associated substrates (Boice and Bouchier-Hayes, 2020). We investigated the influences of TRPM2-AS silencing on the apoptosis of RB cells via determining levels of apoptosis-related proteins, including Bcl-2, Bax, and cleaved caspase-3. The results showed that downregulation of TRPM2-AS significantly increased levels of Bax and cleaved caspase-3 but decreased Bcl-2 expression. Surprisingly, cleaved caspase-3 expression also showed the obvious band in the sh-NC group. This may be because some cells underwent apoptosis, which was verified by flow cytometry. Cell cycle was closely related to the growth of cancer cells; hence, we further analyzed the cell cycle. We found that downregulation of TRPM2-AS significantly attenuated G2/M arrest, which was accompanied by the increase of cyclin B1 and CDK1 and a decrease of p-CDK1. Taken together, these results verified that TRPM2-AS could promote the malignant phenotypes of RB cells.

Studies have demonstrated that lncRNAs, including TRPM2-AS, mainly acted as miRNA sponges, leading to the interference of miRNA functions. For example, TRPM2-AS promoted gastric cancer progression by acting as sponges of miR-612 and miR-195 (Huang et al., 2019; Xiao et al., 2020). TRPM2-AS stimulated proliferation and suppressed apoptosis of breast cancer cells through sponging miR-140-3p to regulate PYCR1 (Sun et al., 2019). We identified that several miRNAs could interact with TRPM2-AS using online bioinformatics tools. miR-497 is a well-known tumor suppressor in multiple cancers, including RB (Li et al., 2017a; Hoareau-Aveilla et al., 2019; Jeong et al., 2020; Li et al., 2020). In this study, we also found miR-497 expression was decreased in RB samples, and there was a negative correlation between miR-497 and TRPM2-AS in these samples. Hence, miR-497 was chosen for further study. The direct binding relationship between TRPM2-AS and miR-497 was confirmed by luciferase activity assay and biotin-labeled miR-497 pull-down analysis. Downregulation of TRPM2-AS upregulated miR-497 expression in both RB cells and mouse tumor tissues. And miR-497 inhibitor reversed the effects of TRPM2-AS silencing on the proliferation, migration, invasion, apoptosis, and cell cycle of RB cells. In general, these data suggested that TRPM2-AS functioned via negatively modulating miR-497 expression in RB.

WEE1 kinase is a key regulator of the G2/M checkpoint that is associated with phosphorylation and inactivation of CDK1, leading to the prevention of entry into mitosis in response to cellular DNA damage (Matheson et al., 2016). Studies have shown that WEE1 was highly expressed in many types of cancers, such as breast cancers, hepatocellular carcinoma, glioblastoma, melanoma (Matheson et al., 2016). Elevated WEE1 has been reported to be associated with tumor progression and poor disease-free survival in some cancers (Matheson et al., 2016). And, the inhibitor of WEE1 has been used in clinical trial designs (Cole et al., 2020; Kong et al., 2020). In this study, we found that WEE1 expression was upregulated in human RB tissues and cells. High expression of WEE1 was involved in the advanced clinical stage and optic nerve invasion in RB patients. We also found downregulation of WEE1 significantly inhibited the proliferation, migration, and invasion, increased apoptosis, and suppressed the G2/M arrest of RB cells. These results indicated that WEE1 was an oncogene in RB. Previous studies have found that miRNAs could regulate the malignant phenotypes of tumor cells via targeting WEE1 (Creevey et al., 2013; Li et al., 2017b). Creevey et al. (Creevey et al., 2013) have verified the direct binding relationship between miR-497 and WEE1 in neuroblastoma. Hence, we further analyzed the association between miR-497 and WEE1 in RB. Biotin-labeled miR-497 pull-down assay confirmed the direct binding between them in RB cells. miR-497 negatively mediated WEE1 expression at both transcriptional and translational levels. In RB tissue samples, there was a negative correlation between WEE1 and miR-497, whereas a positive correlation between WEE1 and TRPM2-AS. In the following, our results showed that knockdown of TRPM2-AS markedly decreased WEE1 expression, which was abrogated by miR-497 inhibitor. And, WEE1 reintroducing abrogated, at least partly, the effects of TRPM2-AS downregulation on the malignant phenotypes of RB cells. These results indicated that TRPM2-AS functioned mainly through regulating WEE1 expression via miR-497.

In conclusion, we found that lncRNA TRPM2-AS was highly expressed and functioned as a tumor promoter gene in RB. Downregulation of TRPM2-AS suppressed the proliferation, migration, and invasion and attenuated G2/M arrest, whereas it promoted apoptosis, which was largely related to the inhibition of WEE1 via sponging miR-497. This study further clarified the molecular mechanism in RB development and TRPM2-AS might be a novel target of RB treatment. However, with the limited sample size, the clinical significance, including the prognostic significance of TRPM2-AS in RB, still needs further research. In addition, about the treatment of targeting TRPM2-AS and WEE1 in RB, we think targeting TRPM2-AS may be more effective because TRPM2-AS can negatively regulate miR-497 expression, which also targets vascular endothelial growth factor A, a gene closely related to cell proliferation, migration, invasion, and tumor angiogenesis (Li et al., 2017a), except for WEE1 in RB.

## Data Availability

The datasets presented in this study can be found in online repositories. The names of the repository/repositories and accession number(s) can be found as follows: https://www.ncbi.nlm.nih.gov/genbank/, NR_109964.1.
