# Supplementary material for: Long Noncoding RNA TRPM2-AS Promotes the Growth, Migration, and Invasion of Retinoblastoma via miR-497/WEE1 Axis
Source: Front Pharmacol. 2021 Apr 12;12:592822. doi: 10.3389/fphar.2021.592822 (PMC8112210; doi:10.3389/fphar.2021.592822)
Supplement: Supplementary file 2 [file Image1.tif]

Frontiers | Long non-coding RNA TRPM2-AS promotes the growth, migration and invasion of retinoblastoma via miR-497/WEE1 axis | Pharmacology


- About
- Journals
- Research Topics
- Articles
- More

Submit

My Frontiers

Office

- TSOF
  - TSOF
  - Article Production

Typesetter 3

frontiersproduction@tnq.co.in

- Profile
- Settings & Privacy
- Help Center
- Logout

Submit

**Impact Factor 4.225** | **CiteScore 5.0**More on impact ›

|  |  |
| --- | --- |
| Frontiers in Pharmacology | Pharmacology of Anti-Cancer Drugs |

Toggle navigation


Section


- (current)Section
- About
- Articles
- Research topics
- For authors 
  - Why submit?
  - Fees
  - Article types
  - Author guidelines
  - Review guidelines
  - Submission checklist
  - Contact editorial office
  - Submit your manuscript
- Editorial board

- *Article alerts*

Articles


**Suggest a Research Topic >**

- 66
  total views

 View Article Impact

**Suggest a Research Topic >**

##### SHARE ON

- Facebook

  0
- Twitter

  0
- LinkedIn

  0
- AddThis

  New


## Original Research ARTICLE

Front. Pharmacol.
| doi: 10.3389/fphar.2021.592822

# Long non-coding RNA TRPM2-AS promotes the growth, migration and invasion of retinoblastoma via miR-497/WEE1 axis Provisionally accepted The final, formatted version of the article will be published soon. **Notify me**

Aipeng Li1, 
Jingpu Yang2, 
Ting Zhang1, 
Lin Li3 and 
 Miyang Li3\*

- 1First Affiliated Hospital of Jilin University, China
- 2Second Affiliated Hospital of Jilin University, China
- 3China-Japan Union Hospital, Jilin University, China

Long non-coding RNAs (lncRNAs) exhibit vital roles in many cancers, including retinoblastoma (RB), the most common primary intraocular malignancy tumor of infancy. A novel lncRNA TRPM2-AS has been demonstrated to be related to multiple cancers, however, its role in RB remains unclear. Here, we aimed to investigate the function of TRPM2-AS in RB. In this study, TRPM2-AS expression in 35 human RB tissues and RB cell lines was detected by real-time PCR. And the relationship between its expression and clinicopathological characteristics of RB patients was analyzed. RB cells’ proliferation, migration, invasion, apoptosis and cell cycle were explored after silencing TRPM2-AS. The mechanism of TRPM2-AS in RB was focused on miR-497/WEE1 axis. Additionally, the role and mechanism of TRPM2-AS were confirmed in a xenograft mouse model. We found TRPM2-AS expression was enhanced in RB tissues and cells. And higher TRPM2-AS expression was related to advanced clinical stage and optic nerve invasion in patients. Downregulation of TRPM2-AS significantly inhibited proliferation, migration and invasion, elevated apoptosis, and attenuated G2/M phase arrest in RB cells, and suppressed tumor growth in vivo. TRPM2-AS acted as a ceRNA for miR-497 to positively regulate WEE1 expression. miR-497 inhibitor or WEE1 overexpression dramatically reversed the effects of TRPM2-AS downregulating on the malignant phenotypes of RB cells. Therefore, TRPM2-AS is an oncogenic lncRNA in RB, and it functions largely through the miR-497/WEE1 pathway. Despite the limited sample size, this study indicates that TRPM2-AS may be a candidate target in RB therapies.

Keywords: 
long non-coding RNA, TRPM2-AS, Retinoblastoma, MiR-497, Wee1

Received: 08 Aug 2020;
Accepted: 10 Feb 2021.

Copyright: © 2021 Li, Yang, Zhang, Li and Li. This is an open-access article distributed under the terms of the Creative Commons Attribution License (CC BY). The use, distribution or reproduction in other forums is permitted, provided the original author(s) and the copyright owner(s) are credited and that the original publication in this journal is cited, in accordance with accepted academic practice. No use, distribution or reproduction is permitted which does not comply with these terms.

\* Correspondence: 
Dr. Miyang Li, China-Japan Union Hospital, Jilin University, Changchun, 130033, Jilin Province, China, miyang@jlu.edu.cn

Write a comment...

Add

##### COMMENTARY

##### ORIGINAL ARTICLE

##### People also looked at

## Inhibition of Shear-Induced Platelet Aggregation by Xueshuantong via Targeting Piezo1 Channel-Mediated Ca2+ Signaling Pathway

Lei Liu, Qiongling Zhang, Shunli Xiao, Zhengxiao Sun, Shilan Ding, Ying Chen, Lan Wang, Xiaojie Yin, Fulong Liao, Lin-Hua Jiang, Mei Xue and Yun You

## Higher ETV5 Expression Associates With Poor 5-Florouracil-Based Adjuvant Therapy Response in Colon Cancer

Anil K. Giri

## Transcriptomic and microRNA expression profiles identify biomarkers for predicting neo-chemoradiotherapy response in esophageal squamous cell carcinomas

Jian Wang, Pengyi Yu, Judong Luo, Zhiqiang Sun, Jingping Yu and Jianlin Wang

## Computational Drug Repositioning and Experimental Validation of Ivermectin in Treatment of Gastric Cancer

Hanne-Line Rabben, Gøran Troseth Andersen, Aleksandr Ianevski, Magnus Kringstad Olsen, Denis Kainov, Jon Erik Grønbech, Timothy Cragin Wang, Duan Chen and Chun-Mei Zhao

## A bibliometrics analysis of metformin development from 1980 to 2019

Yanjun Song, Pei Ma, Yu Gao, Peigen Xiao, Lijia Xu and Haibo Liu

**Suggest a Research Topic >**

×

#### Supplementary Material

  

There is no supplementary material currently available for this article

Loading supplemental data...

  

|  | File Name |  |
| --- | --- | --- |
|  | Table 1.DOCX |  |
|  | Data Sheet 1.XLSX |  |
|  | Image 1.TIF |  |

  

Close

- About Frontiers
- Institutional Membership
- Books
- News
- Frontiers' social media
- Contact
- Careers
- Submit
- Newsletter
- Help Center
- Terms & Conditions
- Privacy Policy

© 2007 - 2021 Frontiers Media S.A. All Rights Reserved

### Privacy Preference Center

Our website uses cookies that are necessary for its operation. Additional cookies are only used with your consent. These cookies are used to store and access information such as the characteristics of your device as well as certain personal data (IP address, navigation usage, geolocation data) and we process them to analyse the traffic on our website in order to provide you a better user experience, evaluate the efficiency of our communications and to personalise content to your interests. Some cookies are placed by third-party companies with which we work to deliver relevant ads on social media and the internet. Click on the different categories' headings to change your cookie preferences. Click on "More Information" if you wish to learn more about how data is collected and shared.
More information

### Manage Consent Preferences

#### Strictly Necessary Cookies

Always Active

These cookies are necessary for the website to function and cannot be switched off in our systems. They are usually only set in response to actions made by you which amount to a request for services, such as setting your privacy preferences, logging in or filling in forms. You can set your browser to block or alert you about these cookies, but some parts of the site will not then work. These cookies do not store any personally identifiable information.

#### Analytics Cookies

Analytics Cookies

These cookies allow us to count visits and traffic sources so we can measure and improve the performance of our site. They help us analyse which pages are the most and least popular and see how visitors move around the site.    All information these cookies collect is aggregated and therefore anonymous.

#### Functional Cookies

Functional Cookies

These cookies enable the website to provide enhanced functionality and personalisation. They may be set by us or by third party providers whose services we have added to our pages. If you do not allow these cookies then some or all of these services may not function properly.

#### Advertising Cookies

Advertising Cookies

These cookies may be set through our site by our advertising partners. They may be used by those companies to build a profile of your interests and show you relevant adverts on other sites.    They do not store directly personal information, but are based on uniquely identifying your browser and internet device. If you do not allow these cookies, you will experience less targeted advertising.

### Back Button Performance Cookies

Vendor Search  Search Icon

Filter Icon

Clear

checkbox label label

Apply Cancel

Consent Leg.Interest

checkbox label label

checkbox label label

checkbox label label

Confirm My Choices
