# Supplementary material for: Long Noncoding RNA TRPM2-AS Promotes the Growth, Migration, and Invasion of Retinoblastoma via miR-497/WEE1 Axis
Source: Front Pharmacol. 2021 Apr 12;12:592822. doi: 10.3389/fphar.2021.592822 (PMC8112210; doi:10.3389/fphar.2021.592822)
Supplement: Supplementary file 3 [file table1.docx]

**Table 1 Correlation between expression of TRPM2-AS/WEE1 and clinicopathological factor in RB patients**

| Clinicopathological features | Number | TRPM2-AS | | |  | WEE1 | | |
| --- | --- | --- | --- | --- | --- | --- | --- | --- |
|  |  | Low  (18) | High  (17) | *P* value |  | Low  (17) | High  (18) | *P* value |
| Age |  |  |  | 0.146 |  |  |  | 0.471 |
| <3 | 25 | 15 | 10 |  |  | 11 | 14 |  |
| ≥3 | 10 | 3 | 7 |  |  | 6 | 4 |  |
| Gender |  |  |  | 0.505 |  |  |  | 0.738 |
| Male | 19 | 11 | 8 |  |  | 10 | 9 |  |
| Female | 16 | 7 | 9 |  |  | 7 | 9 |  |
| Laterality |  |  |  | 0.402 |  |  |  | 0.691 |
| Unilateral | 28 | 13 | 15 |  |  | 13 | 15 |  |
| Bilateral | 7 | 5 | 2 |  |  | 4 | 3 |  |
| Clinical Stage |  |  |  | 0.018^*^ |  |  |  | 0.007^*^ |
| Early stages (A, B) | 9 | 8 | 1 |  |  | 8 | 1 |  |
| Advanced stages (C, D, E) | 26 | 10 | 16 |  |  | 9 | 17 |  |
| Differentiation |  |  |  | 0.50 |  |  |  | 0.092 |
| Well/moderately | 15 | 9 | 6 |  |  | 10 | 5 |  |
| Poorly/undifferentiated | 20 | 9 | 11 |  |  | 7 | 13 |  |
| Optic nerve invasion |  |  |  | 0.002^*^ |  |  |  | 0.035^*^ |
| Negative | 22 | 16 | 6 |  |  | 14 | 8 |  |
| Positive | 13 | 2 | 11 |  |  | 3 | 10 |  |
| Family history |  |  |  | 0.603 |  |  |  | 0.229 |
| Negative | 32 | 17 | 15 |  |  | 17 | 15 |  |
| Positive | 3 | 1 | 2 |  |  | 0 | 3 |  |

^*^*P*<0.05
